# Антоцианы как компоненты функционального питания

**DOI:** 10.18699/VJ21.022

**Published:** 2021-03

**Authors:** R.S. Yudina, E.I. Gordeeva, O.Yu. Shoeva, M.A. Tikhonova, E.K. Khlestkina

**Affiliations:** Institute of Cytology and Genetics of the Siberian Branch of the Russian Academy of Sciences, Novosibirsk, Russia; Institute of Cytology and Genetics of the Siberian Branch of the Russian Academy of Sciences, Novosibirsk, Russia; Institute of Cytology and Genetics of the Siberian Branch of the Russian Academy of Sciences, Novosibirsk, Russia; Institute of Cytology and Genetics of the Siberian Branch of the Russian Academy of Sciences, Novosibirsk, Russia Scientific Research Institute of Physiology and Basic Medicine, Novosibirsk, Russia; Institute of Cytology and Genetics of the Siberian Branch of the Russian Academy of Sciences, Novosibirsk, Russia Federal Research Center the N.I. Vavilov All-Russian Institute of Plant Genetic Resources (VIR), St. Petersburg, Russia

**Keywords:** plants, pigments, secondary metabolites, flavonoids, anthocyanins, regulatory genes, structural genes, antioxidants, biological activity, растения, пигменты, вторичные метаболиты, флавоноиды, антоцианы, регуляторные гены, структурные гены, антиоксиданты, биологическая активность

## Abstract

Среди встречающихся в природе пигментов антоцианы являются, пожалуй, одной из наиболее изученных групп. Начиная с первых исследований о физико-химических свойствах антоцианов, проведенных еще в XVII в. британским естествоиспытателем Р. Бойлем, наука об этих уникальных соединениях сделала огромный шаг вперед. На сегодняшний день достаточно хорошо исследованы структура и функции антоцианов в растительных клетках, а путь их биосинтеза – один из самых полно охарактеризованных путей биосинтеза вторичных метаболитов как на биохимическом, так и на генетическом уровне. Наряду с этими фундаментальными достижениями, мы начинаем осознавать потенциал антоцианов как соединений промышленного значения, как пигментов самих по себе, а также в качестве компонентов функционального питания, способствующих предупреждению и снижению риска развития хронических заболеваний. Долгое время биологическая активность антоцианов была недооценена, в частности, из-за данных об их низкой биодоступности. Однако в ходе исследований было показано, что в организме человека и животных эти соединения активно метаболизируются и биодоступность, оцененная с учетом их метаболитов, превышала 12 %. Экспериментально подтверждено, что антоцианы обладают антиоксидантными, противовоспалительными, гипогликемическими, антимутагенными, антидиабетическими, противораковыми, нейропротекторными свойствами, а также полезны для здоровья глаз. Однако проведенные исследования не всегда могут объяснить молекулярные механизмы действия антоцианов в организме человека. По некоторым данным, наблюдаемые эффекты объясняются действием не антоцианов, а их метаболитов, которые, благодаря своей повышенной биодоступности, могут быть более биологически активными, чем исходные соединения. Высказывается также предположение о положительном эффекте на здоровье человека всего комплекса полифенольных соединений, поступающего в организм в составе растительной пищи. В представленном обзоре суммированы результаты основных направлений исследований антоцианов в качестве компонентов функционального питания. Отдельное внимание уделено результатам генетических исследований синтеза пигментов, данные которых приобретают особую важность в связи с актуализацией селекционных программ, направленных на повышение содержания антоцианов у культурных растений. Ключевые слова: растения; пигменты; вторичные метаболиты; флавоноиды; антоцианы; регуляторные гены; структурные гены; антиоксиданты; биологическая активность.

## Abstract (en)

Among the natural pigments, anthocyanins are assumed to represent one of the most studied groups. Starting with the first studies on the physicochemical properties of anthocyanins carried out in the 17th century by British naturalist Robert Boyle, the science about these unique compounds has progressed substantially. To date, the structure and functions of anthocyanins in plant cells have been well studied, and the pathway of their biosynthesis is one of the most fully characterized pathways of secondary metabolite biosynthesis at both the biochemical and genetic levels. Along with these fundamental achievements, we are beginning to realize the potential of anthocyanins as compounds of industrial importance, as pigments themselves, as well as components of functional food that contribute to the prevention and reduction of risk of chronic diseases. For a long time, the biological activity of anthocyanins has been underestimated, in particular, due to the data on their low bioavailability. However, studies showed that in humans and animals, these compounds are actively metabolized and the bioavailability, estimated taking into account their metabolites, exceeded 12 %. It has been experimentally shown that anthocyanins have antioxidant, anti-inflammatory, hypoglycemic, antimutagenic, antidiabetic, anti-cancer, neuroprotective properties, and they are beneficial for eye health. However, the studies conducted cannot always explain the molecular mechanism of action of anthocyanins in the human body. According to some reports, the observed effects are not due to the action of anthocyanins themselves, but to their metabolites, which can be more biologically active because of their increased bioavailability. Other data ascribe the positive effect on human health not to individual anthocyanins, but to the whole complex of polyphenolic compounds consumed. The review summarizes the results of the studies of anthocyanins as components of functional food. Special attention is paid to genetic control of the pigment synthesis. These data are of particular importance in respect to the initiated breeding programs aimed at increasing the content of anthocyanins in cultural plants.

## Введение

В последние годы в науке о питании появилось новое направление – функциональное питание. Его концепция возникла в Японии в 1980–1990-х гг. и базируется на употреблении в пищу так называемых функциональных продуктов питания (Фотев и др., 2018). Согласно определению, функциональными называют пищевые продукты, содержащие физиологически активные, ценные и безопасные для здоровья ингредиенты с известными физико-химическими характеристиками, для которых выявлены и научно обоснованы полезные для сохранения и улучшения здоровья свойства (ГОСТ Р 52349-2005). К таким веществам относятся растворимые и нерастворимые пищевые волокна, витамины, минеральные вещества, жиры и вещества, сопутствующие жирам, полисахариды, вторичные растительные соединения, про- и пребиотики.

В качестве компонентов функционального питания активно исследуются различные биологически активные соединения, среди которых антоцианы привлекают особое внимание (Calderaro et al., 2020). Эти соединения являются водорастворимыми пигментами, окраска которых, в зависимости от структуры и рН среды, может варьировать от красного и пурпурного до синего цвета. Антоцианы широко представлены в группе покрытосеменных растений и встречаются у некоторых представителей голосеменных, тогда как в других таксонах они отсутствуют (Rausher, 2006). Окрашивая генеративные органы и плоды, антоцианы участвуют в привлечении опылителей и распространителей семян, в вегетативных органах они задействованы в адаптивных реакциях к условиям окружающей среды (Hatier, Gould, 2008). 

К настоящему времени появились убедительные, научно-обоснованные данные о пользе антоцианов для животных и человека, помимо их важной роли в жизни растений. Ингибирование антоцианами различных форм рака, метаболических, сердечно-сосудистых и нейродегенеративных заболеваний было задокументировано как на экспериментальных моделях in vitro и in vivo, так и в клинических и эпидемиологических исследованиях (Тараховский и др., 2013; Li et al., 2017). Ранее предполагалось, что только антиоксидантные свойства антоцианов ответственны за их укрепляющие здоровье эффекты. Однако было показано, что они способны взаимодействовать с регуляторными белками, а также с компонентами сигнальных путей и, таким образом, модулировать физиологические процессы, протекающие в организме человека (Li et al., 2017).

Основные источники антоцианов – темноокрашенные плоды, среди которых ягоды бузины, рябины черноплодной, граната и черники – лидеры по содержанию этих соединений (Ramos et al., 2014). В последнее время в качестве источников антоцианов стали рассматривать более экзотические в этом плане культуры, такие как злаки и картофель, зерно и клубни которых также способны накапливать антоциановые соединения (Payyavula et al., 2013; Zhu, 2018). Несмотря на то что и в зерне, и клубнях антоцианов содержится меньше, чем в ягодах, они также являются привлекательным источниками этих соединений, поскольку характеризуются более длительным хранением, доступностью и повседневным употреблением в пищу, по сравнению с сезонными ягодами и фруктами. Исследования потребительских характеристик изделий, приготовленных из зерна пшеницы, содержащего антоцианы, показали, что они не уступают, а по некоторым параметрам даже превосходят контрольные изделия, не содержащие антоцианы (Bartl et al., 2015; Pasqualone et al., 2015; Хлесткина и др., 2017; Ma et al., 2018).

В связи с большим потенциалом антоцианов в качестве компонентов функционального питания сегодня становятся востребованными знания об их генетическом контроле, которые находят свое применение в селекционных программах, направленных на создание новых сортов культурных растений с повышенным содержанием этих ценных для здоровья человека соединений.

В настоящем обзоре представлены данные о синтезе антоцианов у растений и его генетическом контроле, особое внимание уделено исследованиям антоцианов в качестве функциональных компонентов продуктов питания, в частности их биодоступности и механизмам позитивного действия в организме человека.

## Химическая структура и разнообразие антоцианов

Антоцианы относятся к флавоноидным соединениям, входящим в группу полифенолов. В их структуре выделяют углеводный остаток и неуглеводное основание – агликон. Все флавоноиды, включая антоцианы, имеют общий 15-углеродный скелет C6-C3-C6, который состоит из двух ароматических колец А и В, соединенных С3-фрагментом (рис. 1). Степень окисления С-кольца определяет класс флавоноидов, к которому относится искомое соединение. У антоцианов С-кольцо имеет две двойные связи и несет положительный заряд (ион флавилия). 

**Fig. 1.**
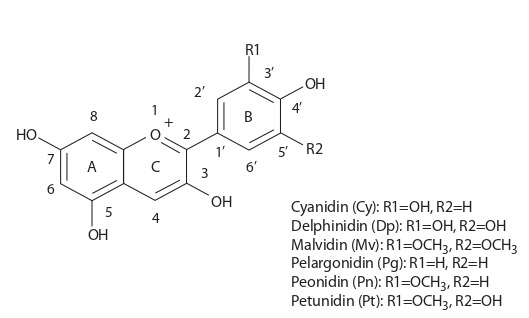
Basic structure of anthocyanins.

Все разнообразие антоцианов, которых, по данным 2006 г., было выявлено около 600 индивидуальных соединений, обуславливают 25 различных агликонов, при этом 90 % идентифицированных антоцианов являются производными только шести из них: цианидина (Cy), дельфинидина (Dp), мальвидина (Mv), пеларгонидина (Pg), пеонидина (Pn) и петунидина (Pt) (Andersen, Jordheim, 2006).

При общем строении С15-углеродного скелета в классе антоцианов индивидуальные соединения выделяют на основе наличия, положения и характера модификаций основного скелета. Все антоциановые соединения представляют собой гликозиды, полученные в результате присоединения сахаров к агликонам, среди которых наиболее частыми являются глюкоза (Glu) и рамноза (Rha), а также встречаются галактоза (Gal), арабиноза (Ara), ксилоза (Xyl), рутиноза (Rut), могут попадаться дисахариды и очень редко – трисахариды. Помимо гликозилирования, антоцианы могут подвергаться ацилированию с помощью ароматических или алифатических ацильных остатков, наиболее распространенными из которых являются п-кумаровая, кофейная и феруловая кислоты. Антоциановые соединения также могут подвергаться метилированию и метоксилированию, а благодаря наличию реакционноспособных гидроксильных групп они легко вступают в реакции алкилирования, образуя эфиры (Запрометов, 1974).

## Биосинтез антоцианов и его генетическая регуляция

Многообразие флавоноидных соединений, включая антоцианы, образуется в результате общего фенилпропаноидного и флавоноидного путей биосинтеза, активность которых зависит от двух групп генов – структурных, кодирующих ферменты биосинтеза, и регуляторных, кодирующих транскрипционные факторы, которые тканеспецифически регулируют экспрессию структурных генов и определяют, таким образом, паттерны распределения пигментов.

**Биосинтез антоцианов**

Биосинтез всех флавоноидных соединений начинается с фенилаланина. Фенилаланин-аммиак-лиаза PAL, циннамат-4-гидроксилаза С4H, 4-кумарат:КоА-лигаза 4CL, действуя поочередно, преобразуют фенилаланин в 4-кумарил-КоА. Последующая конденсация одной молекулы 4-кумарил-КоА и трех молекул малонил-КоА с помощью халконсинтазы CHS приводит к образованию тетрагидроксихалкона и тригидроксихалкона, которые являются предшественниками различных классов флавоноидов и изофлавоноидов соответственно. Под действием халконфлаванонизомеразы CHI тетрагидроксихалкон превращается в нарингенин. Последний служит субстратом для ферментов, осуществляющих реакции гидроксилирования С-кольца в положении С3, либо B-кольца в положении С3′. Так, нарингенин с помощью фермента флаванон3-гидроксилазы F3H преобразуется в дигидрокемпферол DHK, а с помощью флавоноид-3′-гидроксилазы F3′H – в эриодиктиол.

Гидроксилирование DHK с помощью F3′H или флавоноид-3′5′-гидроксилазы F3′5′H приводит к образованию дигидрокверцетина DHQ или дигидромирицетина DHM соответственно. Полученные дигидрофлавонолы DHK, DHQ и DHM восстанавливаются дигидрофлавонол-4- редуктазой, DFR до соответствующих флаван-3,4-диолов лейкопеларгонидина, лейкоцианидина, лейкодельфинидина, которые преобразуются до 3-ОН-антоцианидинов пеларгонидина, цианидина и дельфинидина с помощью фермента антоцианидинсинтазы ANS. Последующие этапы биосинтеза антоцианов относятся к реакциям конечных модификаций, необходимых для их стабилизации и хранения. В этой стадии биосинтеза принимают участие ферменты, относящиеся к классам О-метилтрансфераз ОМТ, гликозилтрансфераз GT и ацилтрансфераз АТ. Антоциановые соединения синтезируются на цитоплазматической поверхности эндоплазматического ретикулума, а затем транспортируются и хранятся в вакуолях (WinkelShirley, 2001).

**Регуляция биосинтеза антоцианов**

В регуляции биосинтеза антоцианов принимают участие транскрипционные факторы, принадлежащие к семействам MYB, bHLH и WD40, которые для выполнения регуляторных функций объединяются в MYB-bHLH-WD40 (MBW) комплексы (Hichri et al., 2011). Их регуляция может быть светозависимой и светонезависимой. Светозависимая регуляция инициируется фоторецепторами при действии света различной длины волны. Центральное место в передаче сигналов от фоторецепторов к синтезу антоцианов занимает регуляторный фактор ELONGATED HYPOCOTYL 5 (HY5) (Bulgakov et al., 2017), который напрямую может связываться с промотором гена PAP1, кодирующего транскрипционный фактор MYB (Shin et al., 2013). Другой важный участник светозависимой регуляции – убиквитинлигаза COP1, мишенями которой служат регуляторные факторы, вовлеченные в биосинтез антоцианов (например, PAP1 и PAP2) (Bulgakov et al., 2017).

У некоторых видов растений синтез антоцианов осуществляется в подземных органах, например в клубнях картофеля, что исключает его светозависимую регуляцию. Хотя точный механизм светонезависимой регуляции неизвестен, существует предположение, что он реализуется посредством сахарозы. Так, в промоторной области гена AN1, кодирующего MYB-подобный транскрипционный фактор, регулирующий синтез антоцианов в клубнях картофеля, у сортов с фиолетовой окраской клубней было выявлено шесть SURE (sucrose responsive elements) элементов, тогда как у сортов с белой и желтой окраской клубней этот ген содержал один SURE. Предположительно, сахароза активирует экспрессию гена AN1, который, в свою очередь, активирует экспрессию структурных генов биосинтеза антоцианов, а также генов, кодирующих ферменты гидролиза сахарозы, такие как синтаза сахарозы и инвертаза. Гидролитические ферменты расщепляют сахарозу, приводя к снижению ее уровня в клетке с высвобождением гексоз, продукты распада которых служат предшественниками для синтеза фенилпропаноидов (Payyavula et al., 2013).

Фенотипическое изменение окраски у растений часто обусловлено мутациями именно в регуляторных генах, которые рассматриваются, таким образом, как наиболее эффективные мишени для селекции и биотехнологии. К примеру, накопление антоцианов в мякоти яблока происходит благодаря усиленной активации гена MdMYB10, в промоторе которого присутствуют пять 23-нуклеотидных повторов (Espley, 2009). Сходным образом накопление большого количества антоцианов в мякоти кровавого апельсина обусловлено инсерцией Copia-подобного ретротранспозона, приводящей к усилению экспрессии близлежащего гена Ruby, кодирующего транскрипционный фактор MYB, регулирующий синтез антоцианов (Butelli et al., 2012). У мягкой пшеницы выявлено шесть 261-нуклеотидных тандемных повторов в промоторе доминантного аллеля bHLH-кодирующего гена Pp3/TaPpb1, активирующего экспрессию структурных генов синтеза антоцианов в перикарпе зерновки, тогда как лишь один такой повтор был обнаружен в рецессивном аллеле у неокрашенных сортов (Shoeva et al., 2014; Jiang et al., 2018). Тандемная дупликация двух первых экзонов, первого интрона и части второго интрона, а также инсерция фрагмента длиной около 11 тыс. нуклеотидов обнаружены в промоторе bHLH-кодирующего гена Kala4 у чернозерных сортов риса, но у белозерных сортов такой дупликации не выявлено (Oikawa et al., 2015). 

Помимо мутаций в промоторных районах регуляторных генов, были описаны мутации, приводящие к сдвигу рамки считывания. Так, у ячменя идентифицирован ген HvMyc2, контролирующий синтез антоцианов в алейроновом слое зерновки ячменя, рецессивные аллели которого имеют однонуклеотидную инсерцию в кодирующей части гена (Strygina et al., 2017). Мутации в генах, кодирующих WD40, менее распространены, поскольку эти гены имеют плейотропные функции, которые не ограничиваются лишь синтезом антоцианов (Zhang, Schrader, 2017).

Таким образом, на сегодняшний день достаточно полно охарактеризованы метаболический путь биосинтеза антоцианов, а также его регуляция. Установлено, что качественный состав антоциановых пигментов определяют ферменты биосинтеза, в то время как распределение пигментов в тканях растений, а также его количество контролируются регуляторными генами. Именно с выявлением триггерных регуляторных генов и их картированием на хромосомах связаны современные постгеномные методы селекции культурных растений с повышенным содержанием антоцианов в зерне.

## Биодоступность антоцианов и их метаболизм в организме человека

Долгое время роль антоцианов в функциональном питании была недооценена, в частности, из-за данных об их низкой биодоступности, которая определяется как отношение части вещества, достигающего системной циркуляции, органов и тканей, к общему количеству потребляемого вещества. По некоторым оценкам, лишь 0.4 % исходного количества употребленных в пищу антоцианов детектировано в плазме крови животных и человека (Manach et al., 2005). Такие низкие концентрации антоцианов не могли объяснить физиологические эффекты, наблюдаемые после их употребления. Усовершенствование методов детекции позволило оценить биодоступность антоцианов с учетом их метаболитов и продуктов взаимодействия. С использованием радиоактивно меченного цианидин-3-гликозида C3G было показано, что не менее 12.38 % метаболитов антоцианов выводится из организма человека в составе мочи и выдыхаемого воздуха, что намного выше биодоступности, оцененной только по содержанию исходных соединений в плазме крови (Czank et al., 2013). Исследование образцов крови и продуктов выделения после однократного употребления 500 мг C3G выявило присутствие в них как интактного соединения, так и его катаболизированных производных, среди которых наиболее представленными были глюкурониды протокатехиновой кислоты и цианидина, их метилированные производные, феруловая, гиппуровая, фенилуксусная и фенилпропионовая кислоты (Czank et al., 2013).

У животных, которых кормили антоцианами, эти соединения были обнаружены практически во всех органах, в том числе в тканях головного мозга, что указывает на их активную абсорбцию и способность преодолевать гематоэнцефалический барьер (Celli et al., 2017; SandovalRamírez et al., 2018). Важно отметить, что при краткосрочном употреблении антоцианов в тканях животных преобладают их исходные формы, а при долгосрочном – метаболиты, что связывают с деятельностью кишечной микробиоты (Sandoval-Ramírez et al., 2018).

Метаболизм антоцианов начинается в ротовой полости, где ряд их частично расщепляется гликозидазами бактериальной микрофлоры до соответствующих агликонов (Kamonpatana et al., 2012) (рис. 2). В желудке происходит первоначальная абсорбция гликолизированных антоцианов через желудочную стенку в кровяное русло воротной вены. Именно абсорбцией из желудка объясняют быстрое повышение концентрации антоцианов в плазме крови сразу после их приема. В транспортировке антоцианов через стенку желудка задействованы билитранслоказы и переносчики глюкозы GLUT1 и GLUT3 (Oliveira et al., 2019). По воротной вене антоцианы попадают в печень и распределяются по гепатоцитам, где они подвергаются глюкуронированию, метилированию и сульфатированию, которые осуществляются ферментами уридин-5-дифосфоглюкуронозил-трансферазой UDPGT, катехол-О-метилтрансферазой COMT и сульфотрансферазой SULT соответственно (Celli et al., 2017). В печени часть антоцианов и продуктов их деградации попадает в желчь и секретируется обратно в просвет кишечника через желчный проток (энтерогепатическая рециркуляция), тогда как другая часть попадает в общий кровоток. 

**Fig. 2.**
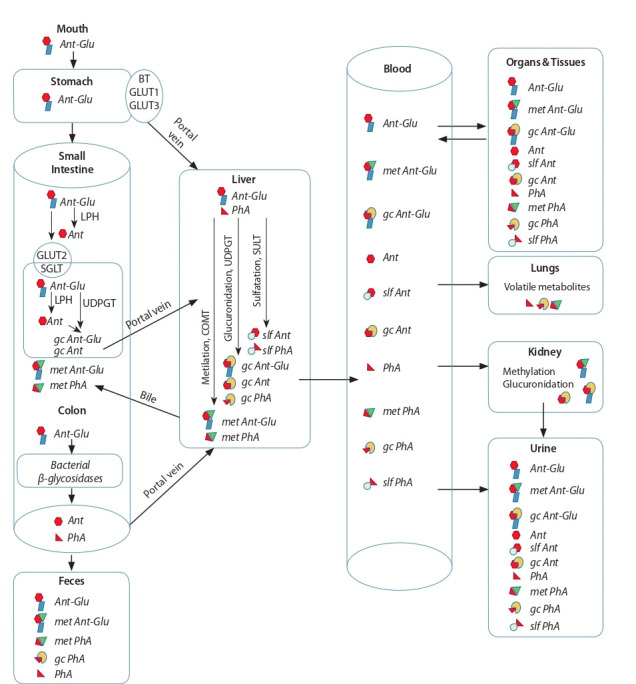
Schematic presentation of the absorption and metabolism of anthocyanins in the human body Ant – anthocyanin aglicon; Ant-Glu – anthocyanin-glucoside; gc Ant – conjugate of anthocyanin aglicon with glucuronic acid; gc Ant-Glu – conjugate of anthocyanin-glucoside with glucuronic acid; gc PhA – conjugate of phenolic acid with glucuronic acid; met Ant-Glu – metilated anthocyanin-glucoside; met PhA – metilated phenolic acid; PhA – phenolic acid; slf Ant – sulfated aglicon of anthocyanin: slf PhA – sulfated phenolic acid; BT – bilitranslocase; COMT – catechol-O-methyltransferase; GLUT 1, 3 – glucose transporters 1, 3; LPH – lactase phlorizin hydrolase; SULT – sulfotransferase; UDPGT – uridine diphosphate-glucuronosyltransferase. After (McGhie, Walton 2007; Celli et al., 2017).

Кроме вышеописанного специфического контура циркуляции, неабсорбированные в желудке антоцианы поступают в кишечник. Тонкий кишечник является вторым участком желудочно-кишечного тракта (ЖКТ), в котором происходит активная абсорбция антоцианов в виде интактных гликозидов либо образующихся под действием гидролаз их агликонов. В клетках энтероцитов каемчатого эпителия кишечника антоцианы, как и другие флавоноиды, могут подвергаться гидролизу под действием лактаза-флоризин гидролазы LPH (Day et al., 2000). Хотя транспортеры антоцианов в клетки кишечника до сих пор точно не установлены, предполагается, что в этом процессе участвуют переносчики глюкозы GLUT2 (Faria et al., 2009) и натрий-зависимый переносчик глюкозы SGLT1 (Zou et al., 2014). В энтероцитах антоцианы и их агликоны глюкуронируются. Перед тем как попасть в кровяное русло, эти вещества по воротной вене доставляются в печень, где они метилируются и сульфатируются соответствующими трансферазами. Антоцианы, не абсорбированные в тонком кишечнике, попадают в толстый кишечник, где они подвергаются расщеплению микробиотой, в результате чего образуются фенольные кислоты и гидроксициннаматы, которые, в свою очередь, могут всасываться эпителием, попадать в кровяное русло и в дальнейшем экскретироваться в мочу (Fang, 2014). Антоцианы и другие флавоноиды не могут быть полностью разрушены микробиотой толстого кишечника, что объясняет присутствие некоторого количества интактных антоцианов в содержимом фекальных выделений (He et al., 2005).

Таким образом, показано, что в организме человека антоцианы активно метаболизируются. Поскольку концентрация и профиль соединений, присутствующих в плазме крови, являются ключевыми для их возможного физиологического воздействия на целевые мишени, существует предположение, что продукты разложения антоцианов в ЖКТ и их конъюгированные метаболиты могут быть более биологически активными, чем исходные антоциановые соединения, вероятно, благодаря своей повышенной биодоступности.

## Роль антоцианов в лечении и профилактике заболеваний

Антоцианы представляют собой общепризнанную догму в народной медицине во всем мире. Антоцианы из различных видов гибискуса исторически использовались в средствах от дисфункции печени и гипертонии, эти соединения из черники имеют давнюю историю применения при микробных инфекциях, диарее и других нарушениях здоровья (Smith et al., 2000; Wang et al., 2000). Именно полифенольными соединениями и их регулярным употреблением в составе красных вин удалось разрешить известный французский парадокс, заключающийся в низкой частоте возникновения ишемической болезни сердца у французов, несмотря на высокий уровень жира в их диете (Renaud, de Lorgeril, 1992). К настоящему времени установлено, что антоцианы характеризуются широким спектром биологического действия в организме человека (Приложение)^1^. Их полезные для здоровья эффекты частично обусловлены антиоксидантными свойствами, а также способностью влиять на регуляторные белки и компоненты сигнальных путей и таким образом модулировать физиологические процессы, протекающие в организме человека.

 Приложение см. по адресу: http://www.bionet.nsc.ru/vogis/download/pict-2021-25/appx5.pdf


**Антоцианы как антиоксиданты**

Наверное, самое известное и активно обсуждаемое свойство антоцианов – их антиоксидантная активность, которая не уступает, а по ряду оценок даже выше, чем у таких общепринятых антиоксидантов, как α-токоферол (Wang et al., 1997), тролокс и катехин (Kähkönen, Heinonen, 2003). Антиоксидантные свойства антоцианов обусловлены их структурными особенностями: числом гидроксильных групп, наличием катехинового фрагмента в В-кольце и иона оксония в С-кольце, паттерном гидроксилирования, метилирования, ацилирования и гликозилирования (Yang et al., 2011). Среди антоциановых агликонов наибольшую антиоксидантную активность проявляют Dp и Cy, за которыми в порядке уменьшения следуют Mv, Pn, Pg и Pt (Lucioli, 2012). 

В организме человека антиоксидантные свойства антоцианов реализуются с помощью их прямого взаимодействия со свободными радикалами (Fukumoto, Mazza, 2000) либо опосредованно через модулирование антиоксидантной защитной системы организма (Shih et al., 2007; Steffen et al., 2008; Toufektsian et al., 2008).

**Антоцианы для здоровья глаз**

Благоприятное влияние антоцианов на улучшение зрения было впервые задокументировано во время Второй мировой войны, когда летчики Королевских военно-воздушных сил Британии для повышения оcтроты зрения в темное время суток употребляли джем из черники (Ghosh, Konishi, 2007). В клинических испытаниях было показано, что употребление антоцианов действительно способствует улучшению дневного, сумеречного и ночного зрения. Однако воздействие антоцианов на зрительные функции наблюдалось не во всех экспериментах, а зависело от принимаемой дозы, ее состава и продолжительности (Nakaishi et al., 2000; Lee et al., 2005).

Одним из механизмов, объясняющих положительное действие антоцианов на зрение, является их способность восстанавливать зрительный пигмент родопсин. При этом было установлено, что гликозид и рутинозид Cy ускоряли регенерацию родопсина, а производные Dp не оказывали никакого воздействия (Matsumoto et al., 2003). В исследовании in vitro было выявлено, что антоцианы также способны ингибировать фотоокисление бисретинола A2E – хромофора липофусциновых гранул, который накапливается с возрастом в эпителиальных клетках сетчатки и может приводить к нарушению целостности их мембран (Jang et al., 2005). Поскольку гибель светочувствительных клеток сетчатки глаза рассматривается как основная причина развития возрастной макулодистрофии, полученные результаты позволяют предположить, что антоцианы могут служить эффективным профилактическим средством этого дегенеративного заболевания.

**Антоцианы для профилактики сердечно-сосудистых заболеваний**

Результаты проведенных исследований указывают на то, что антоцианы помогают предотвратить и частично восстановить нарушения в организме, приводящие к сердечно-сосудистым заболеваниям – группе болезней сердца и кровеносных сосудов, являющихся основной причиной смертности во всем мире (Wallace et al., 2016). В частности, показано, что регулярное употребление антоцианов в пищу снижает концентрацию в плазме липопротеинов низкой плотности, агрегацию тромбоцитов, вероятность развития артериальной гипертензии и эндотелиальной дисфункции (Erlund et al., 2008; Zhu et al., 2014). Благодаря ингибированию ангиотензинпревращающего фермента, активирующего гормон ангиотензин, ответственный за сужение сосудов, антоцианы способствуют снижению артериального давления (Parichatikanond et al., 2012). Помимо этого, антоцианы повышают активность эндотелиальной синтазы оксида азота eNOS и увеличивают, таким образом, высвобождение оксида азота NO, обладающего вазодилатирующими, противотромбозными, антиатерогенными и антипролиферативными свойствами (Xu et al., 2004; Horie et al., 2019).

Благотворное влияние антоцианов на сердечно-сосудистую систему обусловлено также их противовоспалительными и антиагрегатными свойствами. Как противовоспалительные агенты, антоцианы могут подавлять экспрессию генов цитокинов, кодирующих медиаторы воспаления, среди которых ключевое место занимает сигнальный путь ядерного фактора NF-κB (Karlsen et al., 2010). Антоцианы также способны ингибировать экспрессию гена, кодирующего циклооксигеназу-2 COX-2, участвующую в синтезе простагландинов, обладающих противовоспалительной активностью (Hou et al., 2005).

Употребление антоцианов – эффективная профилактика атеросклероза, обусловленного сужением сосудов и снижением кровотока за счет отложения холестерина и некоторых фракций липопротеидов в просвете сосудов (Mauray et al., 2012). Они проявляют свои антиатерогенные свойства благодаря супрессирующему влиянию на образование тромбоцитарных белков хемокинов, участвующих в привлечении циркулирующих лейкоцитов и клеток-предшественников к месту повреждения эндотелия (Song et al., 2014).

Приведенные примеры представляют собой лишь малую часть полученных к настоящему времени данных о благотворном влиянии антоцианов на состояние сердечно-сосудистой системы. На сегодняшний день эта группа соединений рассматривается в качестве эффективных профилактических средств против сердечно-сосудистых патологий.

**Антоцианы для профилактики нейродегенеративных заболеваний**

Богатые антоцианами фрукты могут оказывать положительное влияние на изменение направления старения нейронов и поведения (Joseph et al., 1999). Проведено рандомизированное контролируемое клиническое исследование, в котором люди старшей возрастной группы (70+) с деменцией получали богатый антоцианами вишневый сок (200 мл/день) или контрольный сок с низким содержанием антоцианов. У группы, регулярно получавшей антоцианы, наблюдалось улучшение показателей беглости речи, кратковременной и долговременной памяти (Kent et al., 2017). Было отмечено положительное влияние богатых антоцианами экстрактов шелковицы на индукцию антиоксидантных ферментов и коррекцию когнитивных нарушений у мышей с ускоренным старением и нейродегенерацией альцгеймероподобного типа (Shih et al., 2010). Нейропротекторные эффекты антоцианов могут быть связаны с ослаблением нейротоксичности, индуцируемой перекисью водорода, амилоидом-бета, D-галактозой и ишемией (Tarozzi et al., 2010; Min et al., 2011; Rehman et al., 2017). Показана польза богатого антоцианами зерна пшеницы для когнитивных функций взрослых мышей (Tenditnik et al., 2017). На клеточных моделях болезни Паркинсона выявлено, что экстракты, богатые антоцианами и проантоцианидинами, проявляют нейропротекторную активность против повреждения нейронов нейротоксином ротеноном (Strathearn et al., 2014).

Основными механизмами, с помощью которых антоцианы влияют на функции мозга, служат их способности защищать нейроны от повреждений, индуцированных нейротоксинами и воспалением, активировать синаптическую передачу и улучшать мозговое кровообращение, а также препятствовать высвобождению факторов индукции апоптоза (Spencer, 2010).

**Антиканцерогенные свойства антоцианов**

Антиканцерогенные свойства антоцианов продемонстрированы на клеточных моделях различного типа рака, на экспериментальных животных, а также в ходе клинических наблюдений (Hou, 2003; Smeriglio et al., 2016). Так, снижение жизнеспособности клеток рака гортани, рака желудка и рака молочной железы наблюдалось при обработке их экстрактом из плодов пурумы бразильской (Li et al., 2017). Частота возникновения индуцированных канцерогенами колоректальных аденом и карцином была заметно ниже у крыс, которых кормили фиолетовой кукурузой с высоким уровнем антоцианов по сравнению с группой крыс, в диете которых эти соединения отсутствовали (Hagiwara et al., 2001). 

В ходе клинических наблюдений было установлено, что антоцианы из различных источников способны ингибировать инициацию и прогрессирование рака молочной железы, простаты, печени, толстого и тонкого кишечника, крови, шейки матки, легких, фибросаркомы и метастатической меланомы (Hou, 2003; Smeriglio et al., 2016; Li et al., 2017). При этом следует отметить, что индивидуальные соединения и их композиции проявляют разную антиканцерогенную активность, которая зависит как от типа агликона антоциана, паттерна его гликозилирования, метилирования и ацилирования, так и от комбинации индивидуальных соединений (Smeriglio et al., 2016; Li et al., 2017). Антиканцерогенные свойства антоцианов обусловлены их способностью прерывать клеточный цикл, индуцировать апоптоз, блокировать образование новых сосудов (антиангиогенные свойства), ингибировать окислительное повреждение ДНК, активировать ферменты детоксификации, а также способностью ингибировать циклооксигеназу COX-2 и модулировать иммунный ответ, в том числе через микробиоту (Smeriglio et al., 2016). Перечисленные механизмы могут быть реализованы совместно, что усиливает антиканцерогенные свойства.

**Антоцианы для профилактики метаболических нарушений**

В качестве функциональных компонентов питания антоцианы могут быть использованы для предотвращения ожирения, лечения неалкогольной жировой болезни печени и диабета 2-го типа. Исследования на людях и на экспериментальных животных позволили объяснить молекулярные механизмы, с помощью которых они регулируют жировой и углеводный обмен и снижают резистентность к инсулину (Ghosh, Konishi, 2007; Li et al., 2017).

Употребление антоцианов обуславливает снижение уровня глюкозы в крови как за счет снижения экспрессии генов, кодирующих транспортные белки натрия и глюкозы, GLUT2 и SGLT1 соответственно, в кишечнике, ингибирования кишечной α-глюкозидазы (Adisakwattana et al., 2011) и панкреатической α-амилазы (Sui et al., 2016), так и за счет способности гликозированных антоцианов использовать транспортный механизм глюкозы в эпителиальных клетках желудка, таких как переносчики глюкозы GLUT1 и GLUT3 (Liu et al., 2014; Oliveira et al., 2019). Антоцианы также способны снизить резистентность к инсулину, повышая экспрессию регулируемого инсулином гена белка-транспортера глюкозы GLUT4 путем активации и фосфорилирования альфа-субъединицы АМФактивируемой протеинкиназы АМФКα в белой жировой ткани, скелетных мышцах и печени, стимулирующей поглощение глюкозы и секрецию инсулина β-клетками поджелудочной железы, в то же время ингибируя выработку избытка глюкозы в печени (Tsuda et al., 2004; Takikawa et al., 2010)

Антоцианы оказывают защитное действие на β-клетки поджелудочной железы, уменьшая митохондриальную продукцию активных форм кислорода (Zhang et al., 2010; Sun et al., 2012). Они могут модулировать антиоксидантную защиту, активируя антиокислительные ферменты и способствуя синтезу восстановленного глутатиона (GSH) в печени. Так, потребление антоцианов восстанавливает уровень глутатионпероксидазы 3, который значительно снижается при приеме пищи с высоким содержанием жира (Tsuda, 2016).

При чрезмерном накоплении жировой ткани нарушаются процессы кровоснабжения жировых клеток адипоцитов, появляются очаги некроза и инфильтрация жировой ткани макрофагами, что приводит к избыточному образованию провоспалительных цитокинов и повышению уровня циркулирующих свободных жирных кислот, ведущих к системному воспалению (Tsuda, 2016). Антоцианы улучшают метаболизм жирных кислот и триглицеридов за счет повышения активности липопротеинлипазы в скелетных мышцах (Lefevre et al., 2008). Они также подавляют прирост массы тела, восстанавливают нарушенную функцию печени и значительно увеличивают концентрацию гормона адипонектина, вырабатываемого жировыми клетками, путем активации АМФК, в то же время снижая уровни инсулина и лептина (Takikawa et al., 2010; Wu et al., 2013). Снижая секрецию лептина, вырабатываемого жировой тканью, антоцианы модулируют активность нейропептида Y и рецептора GABAB1 в гипоталамусе, сигнализирующих об энергетическом состоянии тела и контролирующих потребление пищи (Badshah et al., 2013). Другой механизм уменьшения гипергликемии и улучшения чувствительности к инсулину связан с подавлением антоцианами экспрессии ретинол-связывающего белка 4 (Sasaki et al., 2007). Кроме того, антоцианы регулируют FoxO1-опосредованную транскрипцию адипоцитарной триглицеридной липазы и, таким образом, ингибируют липолиз, индуцированный высоким содержанием глюкозы в адипоцитах, что позволяет предположить их потенциальное терапевтическое применение при гиперлипидемии, связанной с диабетом (Guo et al., 2012).

Действие антоцианов на неалкогольную жировую болезнь печени и диабетическую нефропатию также включает снижение накопления липидов и улучшение липидного профиля в печени, ослабление инсулин-резистентности, повышение уровня PPARα, снижение воспаления и окислительного стресса (Takayama et al., 2009; Guo et al., 2012; Qin et al., 2018; Sangsefidi et al., 2019).

**Противомикробные свойства антоцианов**

Антоцианы влияют на микробиоту кишечника. Они способны ингибировать рост патогенных микроорганизмов, таких как Enterococcus spp. и Clostridium perfringens, а также проявляют пребиотические эффекты, ускоряя рост Lactobacillus spp. и Bif idobacterium spp. (Hidalgo et al., 2012). Показано, что экстракты антоцианов повышают проницаемость мембран бактерий, вызывающих пищевые отравления, Listeria monocytogenes, Staphylococcus aureus, Salmonella enteritidis и Vibrio parahaemolyticus. В результате повышенной проницаемости из клеток бактерий происходит утечка белков, нуклеиновых кислот и метаболитов. Помимо этого, антоцианы могут проникать в клетки бактерий и снижать активность ферментов основного метаболизма, таких как щелочная фосфатаза, аденозинтрифосфатаза, а также супероксиддисмутаза, нарушая, таким образом, работу бактериальной клетки (Sun et al., 2018).

## Биологическая значимость природных комплексов, содержащих антоцианы

Как отмечают токсикологи, биологи и практикующие врачи, действие природных соединений ослабляется, когда биологически активные смеси (экстракты) разделяются на очищенные компоненты и вводятся отдельно (Liu, 2003; Lila, Raskin, 2005). Так, было определено, что фитохимические составляющие американской клюквы, хотя и индивидуально эффективные против канцерогенеза человека, обеспечили максимальную защиту только при совместном применении в натуральных смесях (Seeram et al., 2004). В этом исследовании были предложены потенциальные синергетические антипролиферативные эффекты от смесей антоцианов, проантоцианидинов и гликозидов флавонолов. В других исследованиях комбинации двух полифенольных соединений из винограда (ресвератрол и кверцетин) продемонстрировали синергетическую способность индуцировать апоптоз (активацию каспазы-3) в клеточной линии карциномы поджелудочной железы человека (Mouria et al., 2002). Аналогично, смешанный полифенольный экстракт из красного вина показал более сильное ингибирование синтеза ДНК в клетках орального плоскоклеточного рака, чем отдельные соединения, даже когда концентрации индивидуально вводимого кверцетина или ресвератрола были выше, чем концентрации в смешанном экстракте (Elattar, Virji, 1999).

Признанные потенцирующие взаимодействия между компонентами в природном фитохимическом комплексе оказывают сильную поддержку сторонникам «употребления всей (функциональной) пищи», не полагаясь только на однокомпонентные экстракты или вытяжки из пищевых продуктов, которые продаются в форме биологически активных добавок (БАД). В последнем случае потеря взаимодействующих фитохимикатов на этапе разработки продукта может привести к значительному снижению эффективности экстракта. В литературе хорошо задокументировано, что флавоноиды обладают широким спектром биологических свойств (см. Приложение), которые могут объяснять терапевтическое действие смеси взаимодействующих флавоноидов посредством нескольких путей вмешательства одновременно.

Таким образом, в настоящее время представляют огромный интерес не только сами антоциановые пигменты и их польза для здоровья, но и созданные природой растительные продукты, содержащие смеси этих соединений, долгое время применявшиеся в народной медицине без тщательного изучения и научного подтверждения их достоинств.

## Заключение

В представленном обзоре суммированы результаты основных направлений исследований антоцианов в качестве компонентов функционального питания. Потенциальные эффекты, способствующие укреплению здоровья, многогранны. Выявленные положительные эффекты подтверждены экспериментальными исследованиями и клиническими испытаниями. Все эти данные свидетельствуют о том, что регулярное употребление в пищу цветных съедобных плодов, обогащенных антоцианами, и продуктов их переработки способствует улучшению здоровья и качества жизни людей. Отдельное внимание уделено результатам исследований генетического контроля синтеза этих пигментов у растений. Характеристика путей биосинтеза антоцианов различных видов растений и его генетическая регуляция обеспечивают ценный ресурс, позволяющий создавать новые организмы с повышенными функциональными качествами для улучшения питания животных и человека.

## Conflict of interest

The authors declare no conflict of interest.

## References

ГОСТ Р 52349-2005. Продукты пищевые. Продукты пищевые
функциональные. Термины и определения (с Изменением № 1).
Дата введения: 2006-07-01.
[State Standard R 52349-2005. Foodstuffs. Functional Foods. Terms
and Definitions. 2006. (in Russian)]

Запрометов М.Н. Основы биохимии фенольных соединений. М.:
Высш. шк., 1974.
[Zaprometov M.N. Fundamentals of the Biochemistry of Phenolic
Compounds. Moscow: Vysshaya Shkola Publ., 1974. (in Russian)]

Тараховский Ю.С., Ким Ю.А., Абдрасилов Б.С., Музафаров Е.Н.
Флавоноиды: биохимия, биофизика, медицина. Пущино, 2013.
[Tarakhovskiy Yu.S., Kim Yu.A., Abdrasilov B.S., Muzafarov E.N.
Flavonoids: Biochemistry, Biophysics, Medicine. Pushino, 2013. (in
Russian)]

Фотев Ю.В., Пивоваров В.Ф., Артемьева А.М., Куликов И.М., Гончарова Ю.К., Сысо А.И., Гончаров Н.П. Концепция создания
Российской национальной системы функциональных продуктов
питания. Вавиловский журнал генетики и селекции. 2018;22(7):
776-783. DOI 10.18699/VJ18.421.
[Fotev Yu.V., Pivovarov V.F., Artemyeva A.M., Kulikov I.M., Goncharova Y.K., Syso A.I., Goncharov N.P. Concept of producing of
the Russian national system of functional food. Vavilovskii Zhurnal
Genetiki i Selektsii = Vavilov Journal of Genetics and Breeding.
2018;22(7):776-783. DOI 10.18699/VJ18.421. (in Russian)]

Хлесткина Е.К., Усенко Н.И., Гордеева Е.И., Стабровская О.И.,
Шарфунова И.Б., Отмахова Ю.С. Маркер-контролируемое получение и производство форм пшеницы с повышенным уровнем биофлавоноидов: оценка продукции для обоснования
значимости направления. Вавиловский журнал генетики и селекции. 2017;21(5):545-553. DOI 10.18699/VJ17.25-o.
[Khlestkina E.К., Usenko N.I., Gordeeva E.I., Stabrovskaya O.I.,
Sharfunova I.B., Otmakhova Y.S. Evaluation of wheat products
with high flavonoid content: justification of importance of markerassisted development and production of flavonoid-rich wheat cultivars. Vavilovskii Zhurnal Genetiki i Selektsii = Vavilov Journal
of Genetics and Breeding. 2017;21(5):545-553. DOI 10.18699/
VJ17.25-o. (in Russian)]

Adisakwattana S., Yibchok-Anun S., Charoenlertkul P., Wongsasiripat N. Cyanidin-3-rutinoside alleviates postprandial hyperglycemia
and its synergism with acarbose by inhibition of intestinal alphaglucosidase. J. Clin. Biochem. Nutr. 2011;49:36-41. DOI 10.3164/
jcbn.10-116.

Andersen O.M., Jordheim M. The anthocyanins. In: Andersen O.M.,
Markham K.R. (Eds.). Flavonoids: Chemistry, Biochemistry and
Applications. Boca Raton, FL: CRC Press, 2006;452-471.

Badshah H., Ullah I., Kim S.E., Kim T.H., Lee H.Y., Kim M. Anthocyanins attenuate body weight gain via modulating neuropeptide Y
and GABAB1 receptor in rats hypothalamus. Neuropeptides. 2013;
47:347-353. DOI 10.1016/j.npep.2013.06.001.

Bartl P., Albreht A., Skrt M., Tremlová B., Ošťádalová M., Šmejkal K.,
Vovk I., Poklar U.N. Anthocyanins in purple and blue wheat grains
and in resulting bread: quantity, composition, and thermal stability. Int. J. Food Sci. Nutr. 2015;66(5):514-519. DOI 10.3109/0963
7486.2015.1056108.

Bulgakov V.P., Avramenko T.V., Tsitsiashvili G.S. Critical analysis of
protein signaling networks involved in the regulation of plant secondary metabolism: Focus on anthocyanins. Crit. Rev. Biotechnol.
2017;37(6):685-700. DOI 10.3109/07388551.2016.1141391.

Butelli E., Licciardello C., Zhang Y., Liu J., Mackay S., Bailey P., Martin C. Retrotransposons control fruit-specific, cold-dependent accumulation of anthocyanins in blood oranges. Plant Cell. 2012;24(3):
1242-1255. DOI 10.1105/tpc.111.095232.

Calderaro A., Barreca D., Bellocco E., Smeriglio A., Trombetta D.,
Laganà G. Colored phytonutrients: role and applications in the functional foods of anthocyanins. In: Nabavi S.M., Suntar I., Barreca D.,
Khan H. (Eds.). Phytonutrients in Food: From Traditional to Rational Usage. Woodhead Publ., 2020;177-195. DOI 10.1016/B978-0-
12-815354-3.00011-3.

Celli G.B., Ghanem A., Brooks M.S. A theoretical physiologically
based pharmacokinetic approach for modeling the fate of anthocyanins in vivo. Crit. Rev. Food Sci. Nutr. 2017;57(15):3197-3207. DOI
10.1080/10408398.2015.1104290.

Czank C., Cassidy A., Zhang Q., Morrison D.J., Preston T., Kroon P.A.,
Botting N.P., Kay C.D. Human metabolism and elimination of the
anthocyanin, cyanidin-3-glucoside: A (13)C-tracer study. Am. J.
Clin. Nutr. 2013;97:995-1003. DOI 10.3945/ajcn.112.049247.

Day A., Canada F., Diaz J., Kroon P., Mclauchlan R., Faulds C.,
Plumb G.W., Morgan M.R., Williamson G. Dietary flavonoid and
isoflavone glycosides are hydrolysed by the lactase site of lactase
phlorizin hydrolase. FEBS Lett. 2000;468:166-170. DOI 10.1016/
s0014-5793(00)01211-4.

Elattar T.M., Virji A.S. The effect of red wine and its components on
growth and proliferation of human oral squamous carcinoma cells.
Anticancer Res. 1999;19(6B):5407-5414.

Erlund I., Koli R., Alfthan G., Marniemi J. Favorable effects of berry
consumption on platelet function, blood pressure, and HDL cholesterol. Am. J. Clin. Nutr. 2008;87(2):323-331. DOI 10.1093/ajcn/
87.2.323.

Espley R.V. Regulation of anthocyanin accumulation in apple by the
transcription factor MdMYB10. Thesis PhD (Biological Sciences),
University of Auckland, 2009. http://hdl.handle.net/2292/5170
Fang J. Bioavailability of anthocyanins. Drug Metab. Rev. 2014;46:
508-520. DOI 10.3109/03602532.2014.978080.

Faria A., Pestana D., Azevedo J., Martel F., de Freitas V., Azevedo I.,
Mateus N., Calhau C. Absorption of anthocyanins through intestinal
epithelial cells – putative involvement of GLUT2. Mol. Nutr. Food
Res. 2009;53:1430-1437. DOI 10.1002/mnfr.200900007.

Fukumoto L.R., Mazza G. Assessing antioxidant and prooxidant activities of phenolic compounds. J. Agric. Food Chem. 2000;48(8):
3597-3604. DOI 10.1021/jf000220w.

Ghosh D., Konishi T. Anthocyanins and anthocyanin-rich extracts: role
in diabetes and eye function. Asia Pac. J. Clin. Nutr. 2007;16(2):
200-208.

Guo H., Guo J., Jiang X., Li Z., Ling W. Cyanidin-3-O-β-glucoside,
a typical anthocyanin, exhibits antilipolytic effects in 3T3-L1 adipocytes during hyperglycemia: Involvement of FoxO1-mediated transcription of adipose triglyceride lipase. Food Chem. Toxicol. 2012;
50(9):3040-3047. DOI 10.1016/j.fct.2012.06.015.

Hagiwara A., Miyashita K., Nakanishi T., Sano M., Tamano S., Kadota T., Koda T., Nakamura M., Imaida K., Ito N., Shirai T. Pronounced inhibition by a natural anthocyanin, purple corn color,
of 2-amino-1-methyl-6-phenylimidazo[4,5-b]pyridine (PhIP)-associated colorectal carcinogenesis in male F344 rats pretreated with
1,2-dimethylhydrazine. Cancer Lett. 2001;171:17-25. DOI 10.1016/
s0304-3835(01)00510-9.

Hatier J.H.B., Gould K.S. Anthocyanin function in vegetative organs.
In: Winefield C., Davies K., Gould K. (Eds.). Anthocyanins. New
York: Springer, 2008;1-19. DOI 10.1007/978-0-387-77335-3_1.

He J., Magnuson B.A., Giusti M.M. Analysis of anthocyanins in rat intestinal contents: impact of anthocyanin chemical structure on fecal
excretion. J. Agric. Food Chem. 2005;53:2859-2866. DOI 10.1021/
jf0479923.

Hichri I., Barrieu F., Bogs J., Kappel C., Delrot S., Lauvergeat V.
Recent advances in the transcriptional regulation of the flavonoid
biosynthetic pathway. J. Exp. Bot. 2011;62(8):2465-2483. DOI
10.1093/jxb/erq442.

Hidalgo M., Oruna-Concha M.J., Kolida S., Walton G.E., Kallithraka S., Spencer J.P., de Pascual-Teresa S. Metabolism of anthocyanins by human gut microflora and their influence on gut bacterial
growth. J. Agri. Food Chem. 2012;60(15):3882-3890. DOI 10.1021/
jf3002153.

Horie K., Nanashima N., Maeda H. Phytoestrogenic effects of blackcurrant anthocyanins increased endothelial nitric oxide synthase (eNOS)
expression in human endothelial cells and ovariectomized rats.
Molecules. 2019;24(7):1259. DOI 10.3390/molecules24071259.

Hou D.X. Potential mechanisms of cancer chemoprevention by anthocyanins. Curr. Mol. Med. 2003;3(2):149-159. DOI 10.2174/1566524
033361555.

Hou D.X., Yanagita T., Uto T., Masuzaki S., Fujii M. Anthocyanidins
inhibit cyclooxygenase-2 expression in LPS-evoked macrophages:
structure-activity relationship and molecular mechanisms involved.
Biochem. Pharmacol. 2005;70(3):417-425. DOI 10.1016/j.bcp.
2005.05.003.

Jang Y.P., Zhou J., Nakanishi K., Sparrow J.R. Anthocyanins protect
against A2E photooxidation and membrane permeabilization in
retinal pigment cells. Photochem. Photobiol. 2005;81:529-536. DOI
10.1562/2004-12-14-RA-402.

Jiang W., Liu T., Nan W., Jeewani D.C., Niu Y., Li C., Wang Y., Shi X.,
Wang C., Wang J., Li Y., Gao X., Wang Z. Two transcription factors TaPpm1 and TaPpb1 co-regulate anthocyanin biosynthesis in
purple pericarps of wheat. J. Exp. Bot. 2018;69(10):2555-2567. DOI
10.1093/jxb/ery101.

Joseph J.A., Shukitt-Hale B., Denisova N.A., Bielinski D., Martin A.,
McEwen J.J., Bickford P.C. Reversals of age-related declines in
neuronal signal transduction, cognitive, and motor behavioral deficits with blueberry, spinach, or strawberry dietary supplementation.
J. Neurosci. 1999;19(18):8114-8121. DOI 10.1523/JNEUROSCI.
19-18-08114.1999.

Kamonpatana K., Giusti M.M., Chitchumroonchokchai C., MorenoCruz M., Riedl K.M., Kumar P., Failla M.L. Susceptibility of anthocyanins to ex vivo degradation in human saliva. Food Chem. 2012;
135:738-747. DOI 10.1016/j.foodchem.2012.04.110.

Karlsen A., Paur I., Bøhn S.K., Sakhi A.K., Borge G.I., Serafini M.,
Erlund I., Laake P., Tonstad S., Blomhoff R. Bilberry juice modulates plasma concentration of NF-κB related inflammatory markers
in subjects at increased risk of CVD. Eur. J. Nutr. 2010;49(6):345-
355. DOI 10.1007/s00394-010-0092-0.

Kähkönen M.P., Heinonen M. Antioxidant activity of anthocyanins
and their aglycons. J. Agric. Food Chem. 2003;51(3):628-633. DOI
10.1021/jf025551i.

Kent K., Charlton K., Roodenrys S., Batterham M., Potter J., Traynor V.,
Gilbert H., Morgan O., Richards R. Consumption of anthocyaninrich cherry juice for 12 weeks improves memory and cognition in
older adults with mild-to-moderate dementia. Eur. J. Nutr. 2017;56:
333-341. DOI 10.1007/s00394-015-1083-y.

Lee J., Lee H.K., Kim C.Y., Hong Y.J., Choe C.M., You T.W.,
Seong G.J. Purified high-dose anthocyanoside oligomer administration improves nocturnal vision and clinical symptoms in myopia
subjects. Br. J. Nutr. 2005;93:895-899. DOI 10.1079/bjn20051438.

Lefevre M., Wiles J.E., Zhang X., Howard L.R., Gupta S., Smith A.A.,
Ju Z.Y., DeLany J. Gene expression microarray analysis of the effects of grape anthocyanins in mice: a test of a hypothesis-generating paradigm. Metabolism. 2008;57:S52-S57. DOI 10.1016/
j.metabol.2008.03.005.

Li D., Wang P., Luo Y., Zhao M., Chen F. Health benefits of anthocyanins and molecular mechanisms: update from recent decade. Crit.
Rev. Food Sci. Nutr. 2017;57(8):1729-1741. DOI 10.1080/10408398.
2015.1030064.

Lila M.A., Raskin I. Health‐related interactions of phytochemicals.
J. Food Sci. 2005;70(1):R20-R27. DOI 10.1111/j.1365-2621.2005.
tb09054.x.

Liu R.H. Health benefits of fruit and vegetables are from additive and
synergistic combinations of phytochemicals. Am. J. Clin. Nutr. 2003;
78(3):517S-520S. DOI 10.1093/ajcn/78.3.517S.

Liu Y., Li D., Zhang Y., Sun R., Xia M. Anthocyanin increases adiponectin secretion and protects against diabetes-related endothelial
dysfunction. Am. J. Physiol. Endocrinol. Metab. 2014;306(8):E975-
E988. DOI 10.1152/ajpendo.00699.2013.

Lucioli S. Anthocyanins: mechanism of action and therapeutic efficacy.
In: Capasso A. (Ed.). Medicinal Plants as Antioxidant Agents: Understanding Their Mechanism of Action and Therapeutic Efficacy.
Research Signpost. Kerala, India, 2012;27-57.

Ma D., Zhang J., Li Y., Wang C. Quality of noodles made from colourgrained wheat. Czech. J. Food Sci. 2018;36:314-320. DOI 10.17221/
130/2017-CJFS.

Manach C., Williamson G., Morand C., Scalbert A., Rémésy C. Bioavailability and bioefficacy of polyphenols in humans. I. Review of
97 bioavailability studies. Am. J. Clin. Nutr. 2005;81:230S-242S.
DOI 10.1093/ajcn/81.1.243S.

Matsumoto H., Nakamura Y., Tachibanaki S., Kawamura S., Hirayama M. Stimulatory effect of cyanidin 3-glycosides on the regeneration of rhodopsin. J. Agric. Food Chem. 2003;51(12):3560-3563.
DOI 10.1021/jf034132y.

Mauray A., Felgines C., Morand C., Mazur A., Scalbert A., Milenkovic D. Bilberry anthocyanin-rich extract alters expression of genes
related to atherosclerosis development in aorta of apo E-deficient
mice. Nutr. Metab. Cardiovasc. Dis. 2012;22:72-80. DOI 10.1016/
j.numecd.2010.04.011.

McGhie T.K., Walton M.C. The bioavailability and absorption of anthocyanins: towards a better understanding. Mol. Nutr. Food Res.
2007;51:702-713. DOI 10.1002/mnfr.200700092.

Min J., Yu S.W., Baek S.H., Nair K.M., Bae O.N., Bhatt A., Majid A.
Neuroprotective effect of cyanidin-3-O-glucoside anthocyanin in
mice with focal cerebral ischemia. Neurosci. Lett. 2011;500(3):157-
161. DOI 10.1016/j.neulet.2011.05.048.

Mouria M., Gukovskaya A., Jung Y., Buechler P., Hines O., Reber H.,
Pandol S. Food-derived polyphenols inhibit pancreatic cancer
growth through mitochondrial cytochrome C release and apoptosis.
Int. J. Cancer. 2002;98(5):761-769. DOI 10.1002/ijc.10202.

Nakaishi H., Matsumoto H., Tominaga S., Hirayama M. Effects of
black currant anthocyanoside intake on dark adaptation and VDT
work-induced transient refractive alteration in healthy humans.
Altern. Med. Rev. 2000;5(6):553-562.

Oikawa T., Maeda H., Oguchi T., Yamaguchi T., Tanabe N., Ebana K.,
Yano M., Ebitani T., Izawa T. The birth of a black rice gene and its
local spread by introgression. Plant Cell. 2015;27:2401-2414. DOI
10.1105/tpc.15.00310.

Oliveira H., Roma-Rodrigues C., Santos A., Veigas B., Brás N., Faria A., Calhau C., de Freitas V., Baptista P.V., Mateus N., Fernandes A.R., Fernandes I. GLUT1 and GLUT3 involvement in anthocyanin gastric transport-Nanobased targeted approach. Sci. Rep.
2019;9(1):1-14. DOI 10.1038/s41598-018-37283-2.

Parichatikanond W., Pinthong D., Mangmool S. Blockade of the reninangiotensin system with delphinidin, cyanin, and quercetin. Planta
Med. 2012;78:1626-1632. DOI 10.1055/s-0032-1315198.

Pasqualone A., Bianco A.M., Paradiso V.M., Summo C., Gabarcorta G.,
Caponio F., Blanco A. Production and characterization of functional
biscuits obtained from purple wheat. Food Chem. 2015;180:64-70.
DOI 10.1016/j.foodchem.2015.02.025.

Payyavula R.S., Singh R.K., Navarre D.A. Transcription factors, sucrose, and sucrose metabolic genes interact to regulate potato phenylpropanoid metabolism. J. Exp. Bot. 2013;64(16):5115-5131. DOI
10.1093/jxb/ert303.

Qin Y., Zhai Q., Li Y., Cao M., Xu Y., Zhao K., Wang T. Cyanidin3-O-glucoside ameliorates diabetic nephropathy through regulation
of glutathione pool. Biomed. Pharmacother. 2018;103:1223-1230.
DOI 10.1016/j.biopha.2018.04.137.

Ramos P.R., Herrera R., Moya-Leуn M.-A. Anthocyanins: food sources
and benefits to consumer’s health. In: Warner L.M. (Ed.). Handbook
of Anthocyanins: Food Sources, Chemical Applications and Health
Benefits (Biochemistry Research Trends). Hauppauge; New York:
Nova Science Publishers, Inc., 2014.

Rausher M.D. The evolution of flavonoids and their genes. In: Grotewold E. (Ed.). The Science of Flavonoids. New York: Springer,
2006;175-211. DOI 10.1007/978-0-387-28822-2_7.

Rehman S.U., Shah S.A., Ali T., Chung J.I., Kim M.O. Anthocyanins
reversed D-galactose-induced oxidative stress and neuroinflammation mediated cognitive impairment in adult rats. Mol. Neurobiol.
2017;54(1):255-271. DOI 10.1007/s12035-015-9604-5.

Renaud S., de Lorgeril M. Wine, alcohol, platelets, and the French paradox for coronary heart disease. Lancet. 1992;339:1523-1526.

Sandoval-Ramírez B.A., Catalán Ú., Fernández-Castillejo S., Rubió L., Macià A., Solà R. Anthocyanin tissue bioavailability in animals: possible implications for human health. A systematic review.
J. Agric. Food Chem. 2018;66(44):11531-11543. DOI 10.1021/acs.
jafc.8b04014.

Sangsefidi Z.S., Hosseinzadeh M., Ranjbar A.M., Akhondi-Meybodi M., Fallahzadeh H., Mozaffari-Khosravi H. The effect of total
anthocyanin-base standardized (Cornus mas L.) fruit extract on liver
function, tumor necrosis factor α, malondealdehyde, and adiponectin in patients with non-alcoholic fatty liver: a study protocol for a
double-blind randomized clinical trial. Nutr. J. 2019;18(1):39. DOI
10.1186/s12937-019-0465-z.

Sasaki R., Nishimura N., Hoshino H., Isa Y., Kadowaki M., Ichi T.,
Horio F. Cyanidin 3-glucoside ameliorates hyperglycemia and insulin sensitivity due to downregulation of retinol binding protein 4 expression in diabetic mice. Biochem. Pharmacol. 2007;74(11):1619-
1627. DOI 10.1016/j.bcp.2007.08.008.

Seeram N.P., Adams L.S., Hardy M.L., Heber D. Total cranberry extract versus its phytochemical constituents: antiproliferative and
synergistic effects against human tumor cell lines. J. Agri. Food
Chem. 2004;52(9):2512-2517. DOI 10.1021/jf0352778.

Shih P.H., Chan Y.C., Liao J.W., Wang M.F., Yen G.C. Antioxidant and
cognitive promotion effects of anthocyanin-rich mulberry (Morus
atropurpurea L.) on senescence-accelerated mice and prevention
of Alzheimer’s disease. J. Nutr. Biochem. 2010;21(7):598-605. DOI
10.1016/j.jnutbio.2009.03.008.

Shih P.H., Yeh C.T., Yen G.C. Anthocyanins induce the activation of
phase II enzymes through the antioxidant response element pathway
against oxidative stress-induced apoptosis. J. Agric. Food Chem.
2007;55(23):9427-9435. DOI 10.1021/jf071933i.

Shin D.H., Choi M.G., Kim K., Bang G., Cho M., Choi S.-B., Choi G.,
Park Y.-I. HY5 regulates anthocyanin biosynthesis by inducing the
transcriptional activation of the MYB75/PAP1 transcription factor
in Arabidopsis. FEBS Lett. 2013;587:1543-1547. DOI 10.1016/
j.febslet.2013.03.037.

Shoeva O.Y., Gordeeva E.I., Khlestkina E.K. The regulation of anthocyanin synthesis in the wheat pericarp. Molecules. 2014;19(12):
20266-20279. DOI 10.3390/molecules191220266.

Smeriglio A., Barreca D., Bellocco E., Trombetta D. Chemistry, pharmacology and health benefits of anthocyanins. Phytother. Res. 2016;
30(8):1265-1286. DOI 10.1002/ptr.5642.

Smith M., Marley K., Seigler D., Singletary K., Meline B. Bioactive
properties of wild blueberry fruits. J. Food Sci. 2000;65:352-356.
DOI 10.1111/j.1365-2621.2000.tb16006.x.

Song F.L., Zhu Y.N., Shi Z.Y., Tian J.J., Deng X.J., Ren J., Andrews M.C., Ni H.Y., Ling W.H., Yang Y. Plant food anthocyanins
inhibit platelet granule secretion in hypercholesterolaemia: involving the signaling pathway of PI3K-Akt. Thromb. Haemost. 2014;
112:981-991. DOI 10.1160/TH13-12-1002.

Spencer J.P.E. The impact of fruit flavonoids on memory and cognition. British J. Nutr. 2010;104:S40-S47. DOI 10.1017/S000711451
0003934.

Steffen Y., Gruber C., Schewe T., Sies H. Mono-O-methylated flavanols and other flavonoids as inhibitors of endothelial NADPH oxidase. Arch. Biochem. Biophys. 2008;469:209-219. DOI 10.1016/
j.abb.2007.10.012.

Strathearn K.E., Yousef G.G., Grace M.H., Roy S.L., Tambe M.A.,
Ferruzzi M.G., Rochet J.C. Neuroprotective effects of anthocyaninand proanthocyanidin-rich extracts in cellular models of Parkinson’s disease. Brain Res. 2014;1555:60-77. DOI 10.1016/j.brainres.
2014.01.047.

Strygina K.V., Börner A., Khlestkina E.K. Identification and characterization of regulatory network components for anthocyanin synthesis
in barley aleurone. BMC Plant Biol. 2017;17(1):184. DOI 10.1186/
s12870-017-1122-3.

Sui X., Zhang Y., Zhou W. In vitro and in silico studies of the inhibition activity of anthocyanins against porcine pancreatic α-amylase. J. Funct. Foods. 2016;21:50-57. DOI 10.1016/j.jff.2015.11.
042.

Sun C.D., Zhang B., Zhang J.K., Xu C.J., Wu Y.L., Li X., Chen K.S.
Cyanidin-3-glucoside-rich extract from Chinese bayberry fruit protects pancreatic β cells and ameliorates hyperglycemia in streptozotocin-induced diabetic mice. J. Med. Food. 2012;15(3):288-298.
DOI 10.1089/jmf.2011.1806.

Sun X.-H., Zhou T.-T., Wei C.-H., Lan W.-Q., Zhao Y., Pan Y.-J.,
Wu V.C.H. Antibacterial effect and mechanism of anthocyanin
rich Chinese wild blueberry extract on various foodborne pathogens. Food Control. 2018;94:155-161. DOI 10.1016/j.foodcont.
2018.07.012.

Takayama F., Nakamoto K., Kawasaki H., Mankura M., Egashira T.,
Ueki K., Mori A. Beneficial effects of Vitis coignetiae Pulliat leaves
on nonalcoholic steatohepatitis in a rat model. Acta Med. Okayama.
2009;63(2):105-111. DOI 10.18926/AMO/31835.

Takikawa M., Inoue S., Horio F., Tsuda T. Dietary anthocyanin-rich bilberry extract ameliorates hyperglycemia and insulin sensitivity via
activation of AMP-activated protein kinase in diabetic mice. J. Nutr.
2010;140:527-533. DOI 10.3945/jn.109.118216.

Tarozzi A., Morroni F., Merlicco A., Bolondi C., Teti G., Falconi M.,
Hrelia P. Neuroprotective effects of cyanidin 3-O-glucopyranoside
on amyloid beta (25–35) oligomer-induced toxicity. Neurosci. Lett.
2010;473(2):72-76. DOI 10.1016/j.neulet.2010.02.006.

Tenditnik M.V., Tikhonova M.A., Pavlov K.S., Amstislavskaya T.G.,
Khlestkina E.K. Evaluating the neuroprotective potential of wheat
grain with high anthocyanin content in correction of behavioral
deficits induced by amyloid-beta neurotoxicity in mice. In: Belyaev
conference: A triumphant event in commemoration of the centenary
of the birth of Academician Dmitri Belyaev (August 7–10, 2017,
Novosibirsk, Russia): Abstracts. Novosibirsk, 2017.

Toufektsian M., Lorgeril M.D., Nagy N. Chronic dietary intake of
plant-derived anthocyanins protects the rat heart against ischemiareperfusion injury. J. Nutr. 2008;138:747-752. DOI 10.1093/jn/138.
4.747.

Tsuda T. Recent progress in anti-obesity and anti-diabetes effect of berries. Antioxidants. 2016;5(2):13. DOI 10.3390/antiox5020013.

Tsuda T., Ueno Y., Aoki H., Koda T., Horio F., Takahashi N., Kawada T., Osawa T. Anthocyanin enhances adipocytokine secretion and
adipocyte-specific gene expression in isolated rat adipocytes. Biochem. Biophys. Res. Commun. 2004;316:149-157. DOI 10.1016/
j.bbrc.2004.02.031.

Wallace T.C., Slavin M., Frankenfeld C.L. Systematic review of anthocyanins and markers of cardiovascular disease. Nutrients. 2016;
8(1):32-45. DOI 10.3390/nu8010032.

Wang C.J., Wang J.M., Lin W.L., Chu C.Y., Chou F.P., Tseng T.H. Protective effect of Hibiscus anthocyanins against tert-butyl hydroperoxide-induced hepatic toxicity in rats. Food Chem. Toxicol. 2000;
38(5):411-416. DOI 10.1016/S0278-6915(00)00011-9.

Wang H., Cao G., Prior R.L. Oxygen radical absorbing capacity of
anthocyanins. J. Agric. Food Chem. 1997;45(2):304-309. DOI
10.1021/jf960421t.

Winkel-Shirley B. Flavonoid biosynthesis. A colorful model for genetics, biochemistry, cell biology, and biotechnology. Plant Physiol.
2001;126(2):485-493. DOI 10.1104/pp.126.2.485.

Wu T., Qi X., Liu Y., Guo J., Zhu R., Chen W., Yu T. Dietary supplementation with purified mulberry (Morus australis Poir) anthocyanins suppresses body weight gain in high-fat diet fed C57BL/6 mice. Food Chem. 2013;141(1):482-487. DOI 10.1016/j.foodchem.
2013.03.046.

Xu J.W., Ikeda K., Yamori Y. Upregulation of endothelial nitric oxide
synthase by cyanidin-3-glucoside, a typical anthocyanin pigment.
Hypertension. 2004;44:217-222. DOI 10.1161/01.HYP.0000135868.
38343.c6.

Yang M., Koo S.I., Song W.O., Chun O.K. Food matrix affecting anthocyanin bioavailability: review. Curr. Med. Chem. 2011;18(2):291-
300. DOI 10.2174/092986711794088380.

Zhang B., Kang M., Xie Q., Xu B., Sun C., Chen K., Wu Y. Anthocyanins from Chinese bayberry extract protect β cells from oxidative
stress-mediated injury via HO-1 upregulation. J. Agric. Food Chem.
2010;59(2):537-545. DOI 10.1021/jf1035405.

Zhang B., Schrader A. TRANSPARENT TESTA GLABRA 1-dependent regulation of flavonoid biosynthesis. Plants. 2017;6(4):65. DOI
10.3390/plants6040065.

Zhu F. Anthocyanins in cereals: сomposition and health effects. Food
Res. Int. 2018;109:232-249. DOI 10.1016/j.foodres.2018.04.015.

Zhu Y., Huang X., Zhang Y., Wang Y., Liu Y., Sun R., Xia M. Anthocyanin supplementation improves HDL-associated paraoxonase 1
activity and enhances cholesterol efflux capacity in subjects with
hypercholesterolemia. J. Clin. Endocrinol. Metab. 2014;99(2):561-
569. DOI 10.1210/jc.2013-2845.

Zou T.-B., Feng D., Song G., Li H.-W., Tang H.-W., Ling W.-H. The
role of sodium-dependent glucose transporter 1 and glucose transporter 2 in the absorption of cyanidin-3-O-beta-glucoside in Caco-2
cells. Nutrients. 2014;6(10):4165-4177. DOI 10.3390/nu6104165.

